# Analysis of VOCs in Liquids through Vaporization in a Tubular Oven Monitored by Chemical Ionization Mass Spectrometry

**DOI:** 10.3390/s24041048

**Published:** 2024-02-06

**Authors:** Taous Abar, Hélène Mestdagh, Michel Heninger, Joel Lemaire

**Affiliations:** Institut de Chimie Physique, Centre National de la Recherche Scientifique, Université Paris-Saclay, 91400 Orsay, France; taous.abar@universite-paris-saclay.fr (T.A.); helene.mestdagh@universite-paris-saclay.fr (H.M.); joel.lemaire@universite-paris-saclay.fr (J.L.)

**Keywords:** volatile organic compounds, tubular oven, Fourier Transform Ion Cyclotron Resonance, real-time analysis, mass spectrometry, proton transfer reaction

## Abstract

The analysis of chemical compounds present at trace levels in liquids is important not only for environmental measurements but also, for example, in the health sector. The reference technique for the analysis of Volatile Organic Compounds (VOCs) in liquids is GC, which is difficult to use with an aqueous matrix. In this work, we present an alternative technique to GC to analyze VOCs in water. A tubular oven is used to completely vaporize the liquid sample deposited on a gauze. The oven is heated in the presence of a dinitrogen flow, and the gas is analyzed at the exit of the oven by a chemical ionization mass spectrometer developed in our laboratory. It is a low magnetic field Fourier Transform Ion Cyclotron Resonance (FT-ICR) optimized for real-time analysis. The Proton Transfer Reaction (PTR) used during the Chemical Ionization event results in the selective ionization of the VOCs present in the gas phase. The optimization of the desorption conditions is described for the main operating parameters: temperature ramp, liquid quantity, and nitrogen flow. Their influence is studied using a 100 ppmv aqueous toluene solution. The analytical method is then tested on a mixture of seven VOCs.

## 1. Introduction

We present in this paper a new technique for quantitative analysis of Volatile Organic Compounds (VOCs) present in liquids. It is designed for samples available in limited amounts and/or containing a mixture of VOCs at low concentrations. Beyond the instrumental work presented here, the long-term objective of this work is to analyze VOCs in sweat and identify sets of pathologic biomarkers.

Most measurements of VOCs in liquid are performed using GC [1,2,3,4,5,6,7]. It has the advantage of high sensitivity and can be operated on small volumes. GC analysis of VOCs in an aqueous solution is particularly difficult since water injection in a GC column may cause damage to the stationary phase. The analysis requires a time of the order of 20–30 min to perform the chromatographic separation. Moreover, for trace analysis of VOCs in aqueous solutions, sample preparation pre-concentration steps are needed to enrich the analytes, remove water, and replace it with a more suitable organic solvent for GC systems [8,9]. The most common VOC pre-concentration methods for GC analysis are Liquid–Liquid Extraction (LLE); Solid-Phase Extraction (SPE), which requires less solvent than LLE; and membrane extraction techniques [10]. Concerning the ionization method, Atmospheric Pressure Chemical Ionization (APCI) combined either with GC-MS [11] or with LC-MS [12,13] gives good results for the detection of VOCs in liquid samples [14,15]. Different types of mass spectrometers can be used, such as quadrupoles with unit mass resolution or high-resolution Time Of Flight (TOF) mass spectrometers [14,16,17], which allow a better identification of the compounds present and can separate quasi-isobaric ions. GC can also be combined with Ion Mobility Spectrometry (IMS) mass spectrometers [18]. Their resolution is more limited, but they may separate isomers and are good candidates for the development of point-of-care analyzers.

Mass spectrometry analysis of complex VOC mixtures is best performed by Chemical Ionization (CI) methods, allowing soft ionization of the analytes. For online monitoring of VOCs, Proton Transfer Reaction Mass Spectrometry (PTR-MS) was developed by Lindinger et al. [19]. PTR-MS was then widely used to detect mixtures of VOCs at trace levels in the air with applications in many fields [20], such as atmospheric chemistry, environmental research, and plant studies. The first PTR-MS commercialized instruments were based on quadrupoles [21]. To improve mass resolution, PTR-MS was coupled with Time Of Flight Mass Spectrometers (PTR-TOF) [22,23] and Fourier Transform Ion Cyclotron Resonance (PTR-FT-ICR) [24]. A low-field FT-ICR mass spectrometer was developed at Institut de Chimie Physique (ICP) in Orsay, and an industrial version was commercialized by AlyXan [25,26]. The number of papers reporting applications of PTR-MS for the detection of VOCs in liquids is much more limited than in gases. The use of Membrane Inlet Mass Spectrometry (MIMS) associated with a PTR-FT-ICR has been described by Louarn et al. [27,28] and Roumiguières et al. [29]. In this method, the hydrophobic membrane acts both as a physical barrier between the sample solution and the mass spectrometer and as a VOC concentrator. This technique shows good sensitivity, down to the ppb level, for nonpolar compounds in the sample solution. However, the technique has two drawbacks: a large volume of sample solution is needed, and prior determination of the membrane enrichment factor is required for each analyte.

The detection of VOCs in liquids has also been performed by headspace analysis, for example, for the analysis of the VOCs present in alcoholic beverages [30,31,32] and in olive oils [33]. Equilibration between the liquid and the gas phase by bubbling at controlled temperatures enabled the measurement of partition coefficients [34,35].

In this work, a tubular oven was coupled to a low-field CI-FT-ICR mass spectrometer for the analysis of small samples of VOCs in liquids. The coupling was optimized using solutions of toluene in water, and its performances were evaluated on a mixture of seven VOCs in water. This paper describes this new coupling and presents its analytical performance.

## 2. Materials and Methods

### 2.1. Chemical Reagents and Materials

Toluene (≥99.5%) was provided by VWR chemicals (Fontenay-sous-Bois, France). Methanol, ethanol, acetone, 2-butanone, 2-pentanone, 2-hexanone, and 1,4-dioxane HPLC grade (≥98%) were ordered from Sigma-Aldrich (St. Louis, MO, USA). Non-woven gauzes (7.5 cm × 7.5 cm) were purchased from ABSO, laboratory Marque Verte (Villers-lès-Nancy, France).

### 2.2. Chemical Ionization Fourier Transform Ion Cyclotron Resonance Mass Spectrometer

#### 2.2.1. General Description of the CI-FT-ICR Mass Spectrometer

The permanent magnet FT-ICR-MS built in our laboratory was specifically designed for real-time monitoring of VOCs. The magnetic field is generated by a permanent magnet. The magnet is a magnetic assembly called a “Halbach cylinder”. It is made of 3 cylinders of 16 neodymium iron boron (NdFeB) magnetized segments generating a dipolar magnetic field in the bore of the cylinder. The bore is 52 mm in diameter, and the ICR cell is placed in its center. The nominal magnetic field is 1.26 T, with a relative homogeneity of 3 × 10^−3^ over a 1.5 cm diameter spherical volume.

**Figure 1:**
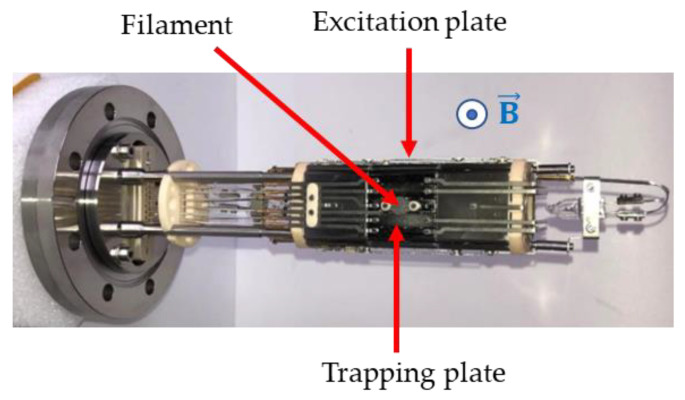
The ICR cell. The direction of the magnetic field $\;\vec{B}$, shown in blue, is perpendicular to the image.

A new ICR cell was designed in our laboratory and is shown in Figure 1. It is derived from a cubic three-section cell in which two of the plates have been replaced by lateral sections made of four electrodes. The central section, in which ions are produced, react, and are detected, has 3 cm × 3 cm × 3 cm internal dimensions. The same two electrodes of the central section are used both for excitation of the ion’s cyclotron motion and for detection: a fast switch connects them to the excitation circuit and then to the detection amplifier. The trapping plates of the central section are divided into five segments on which a superposition of the trapping potential and the excitation potential is applied, resulting in a homogeneous excitation field. The open structure of the two lateral sections ensures a good pumping of the cell. The vacuum chamber containing the cell is pumped by a turbomolecular pump with a residual pressure of 10^−8^ mbar. For the ionization process, an electron beam is used. A filament coated with rhenium-yttria oxide alloy is placed on the side of one of the two central trapping plates. The electrons are produced by thermo emission, and the electron beam is pulsed using an intermediate electrode placed between the filament and the trapping plate. The polarization of the filament defines the energy of the electron beam in the cell (70 eV in this study). To heat the cell and its surroundings, halogen lamps are used. They are placed on both sides of the cell, in front of the openings of the lateral sections.

The ICR cell is used successively for the production of the reactant ions, as a reaction chamber, and finally, as an analyzer in which the ions are detected. The ions formed in the ICR cell are trapped by the combination of the magnetic and electrostatic fields and can stay there for several seconds. The neutral gases needed for the production of the precursor ions or during the chemical ionization of the sample are introduced when needed using pulsed valves and are then pumped away.

The resolution power of the mass spectrometer is higher than 10,000 at *m*/*z* 19. This makes it possible to separate quasi-isobaric compounds and to attribute a chemical formula to the VOCs detected. The precision of the mass measurements is 0.005 u.

The implementation of chemical ionization in an ion trap has many advantages. Different precursor ions can be produced using different neutral gases. In this study, H_3_O^+^ ion precursors were used, and they were produced by electron impact on a pulse of water vapor. To ensure that a pure H_3_O^+^ ion packet is used, all unwanted ions can be ejected. Then, the H_3_O^+^ ions are used for the chemical ionization event, and the VOCs present in the sample are ionized by proton transfer with almost no fragmentation.

#### 2.2.2. Sampling

The gases are pulsed in the instrument, and different gas inlets are available. The sampling using three-way valves has been described in detail by Heninger et al. [25]. For real-time analysis, the instrument must be able to follow a concentration change in the sample. The response time and temporal resolution of the measurements are therefore important factors in the instrument’s performance.

In the present work, one of these inlets is used for introduction of water necessary to produce H_3_O^+^ precursor ion. A metal vial containing water is prepared and degassed through several freeze–pump–thaw cycles. It is then introduced in the cell using a leak valve followed by a three-way valve. The leak valve is adjusted so that when the flow is directed continuously in the ICR chamber, the pressure measured is 2 × 10^−6^ mbar.

**Figure 2:**
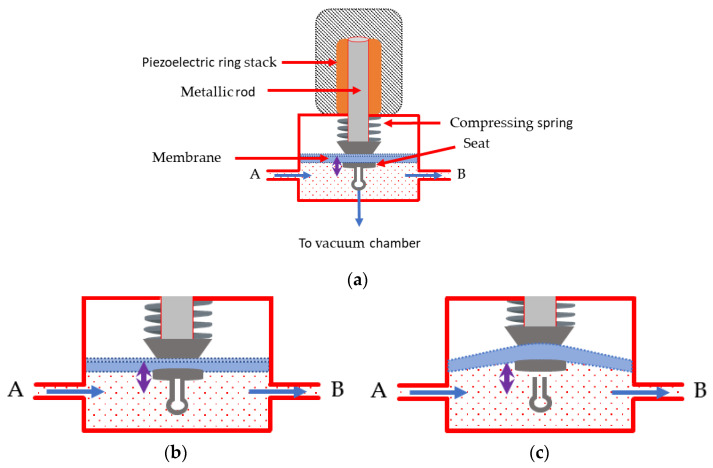
(**a**) Scheme of the piezoelectric valve. The sample gas flows through the valve from inlet (A) to outlet (B). (**b**) Zoom showing the situation when no voltage is applied to the piezoelectric ring stack (valve closed) and (**c**) when voltage is applied to the piezoelectric ring stack (valve open).

Also, a new two-way piezoelectric valve recently developed in collaboration with AlyXan was used. This valve, shown in Figure 2a, allows a direct transfer of small gas samples from atmospheric pressure to the high-vacuum chamber of the mass spectrometer.

With zero voltage applied to the piezoelectric ring stack (abbreviated to “piezo” in the following), the spring pushes on the membrane and the seat, hermetically pressing the seat against the tube opening leading to the vacuum chamber. The gas then flows from A to B, and the valve is closed (Figure 2b). If a voltage is applied, the piezo expands, lifting the rod and opening the gas path to the vacuum. In this configuration, the valve is open, and part of the gas is drawn into the vacuum chamber of the mass spectrometer (Figure 2c).

For real-time measurements, the response time and time resolution of the pulsed sampling are important. The expected performance of the two-way valve lies in its airtightness and in the speed of its temporal response so as to deliver gas pulses with adjustable duration into the vacuum chamber at a pressure in the range of 10^−6^ to 10^−4^ mbar in a controlled and reproducible way.

A suitable parameter for testing this performance is the quantity Pxt, measured by the mass spectrometer and proportional to the amount of gas introduced during a pulse (see Section 2.4.4, Data Analysis and Analyte Quantification). At constant temperature, Pxt depends on two parameters: (i) the voltage applied to the piezo and (ii) the gas pulse duration. The voltage applied to the piezo does not lead to a linear variation of Pxt, and Pxt varies rapidly in a limited potential range. For a given experiment, it is therefore preferable to fix the applied voltage and vary the valve’s opening time.

The present study is performed with a voltage applied to the piezoelectric stack of 70 V. Under these conditions, Pxt varies quite linearly with the valve’s opening time. In the range of 10–300 ms, a linear curve is observed for the dependence of Pxt with opening time.

#### 2.2.3. Operating Sequence

**Figure 3:**
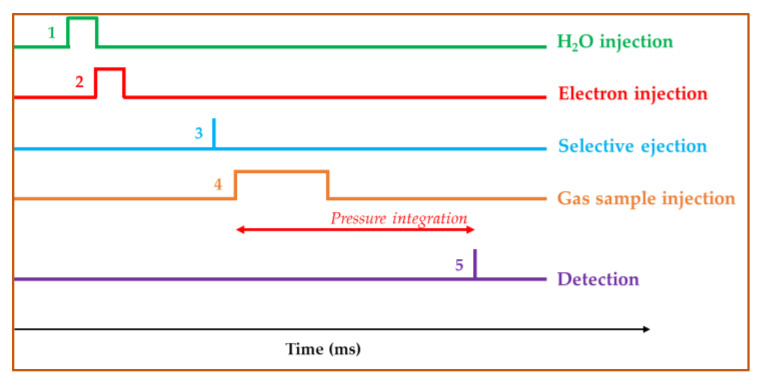
Typical timing of the FT-ICR analytical sequence. The numbers refer to the experimental events described in the text.

The analytical sequence used is shown in Figure 3. (1) A 10 ms pulse of H_2_O gas is introduced in the ICR cell at a pressure of 2 × 10^−6^ mbar. (2) It is ionized by a 10 ms 70 eV electron beam pulse. All the ions produced (H_2_O^+^, OH^+^…) further react with H_2_O to produce H_3_O^+^ ions. (3) If some unwanted ions are present in the cell, they are ejected. (4) The gas sample is introduced in the ICR cell at a pressure of ~10^−5^ mbar, and the VOCs present are ionized by proton transfer reaction (PTR) from H_3_O^+^.

$$H_{3}O^{+}+A\to H_{2}O+AH^{+}$$

The pressure in the cell is continuously monitored by a Bayard–Alpert ionization gauge having a time response close to 1 ms. The sample gas pressure is integrated over time from the beginning of the gas sample pulse up to the detection event (5), giving ion intensities and Pxt values (in 10^−4^ Torr·ms) that are used for the VOCs quantification. At the time of detection, the pressure in the ICR cell is back to values lower than 10^−7^ Torr. The total duration of the sequence is ca. 2500 ms.

### 2.3. Tubular Oven

A tubular oven is used to vaporize the liquid sample. A Model “TF1 11/32/150” was purchased from Carbolite Gero, VERDER Scientific (Eragny sur Oise, France). This oven can heat samples up to 1100 °C. It is fitted with a quartz tube with an external diameter of 32 mm, an internal diameter of 28 mm, and a length of 50 cm. Heating resistors inside the oven ensure that the central part of the quartz tube is heated homogeneously. This central section is 15 cm long. Heat shields are placed inside the quartz tube that isolate the central part from lateral parts. The ends of the quartz tube are fitted with Teflon seals and metal fittings to ensure good system sealing. They also provide connections with the tubes, allowing a circulation of carrier gas through the oven. An additional connection is present only on one of the metal fittings. It is used to insert a type K thermocouple for temperature measurements inside the quartz tube. The thermocouple is connected to a USB interface (USB-TC01 from National Instruments, Austin, TX, USA). The oven is also equipped with a thermocouple that gives a temperature measurement external to quartz tube but close to its center. The internal control of the oven temperature is performed by a model 3016 controller (manufactured by Eurotherm, Worthing, UK), which is interfaced with the computer monitoring the experiment.

### 2.4. Coupling the Tubular Oven with the CI-FT-ICR Mass Spectrometer

#### 2.4.1. General Description of the Experimental Method

**Figure 4:**
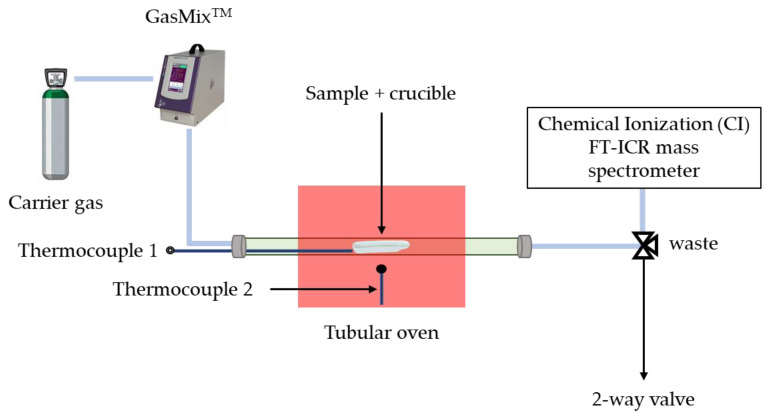
Scheme of the experimental setup.

Figure 4 gives an overview of the experimental setup. The samples are introduced into the tubular oven by placing the gauze soaked with the sample solution in a ceramic combustion boat. A stream of nitrogen controlled using GasMix™ instrument (AlyTech, Juvisy-sur-Orge, France) flows through the oven. The nitrogen pressure in the oven is close to 1 bar. Only a small fraction of the gas flow is introduced in the mass spectrometer; most of the flow is sent to waste. To avoid condensation of VOCs or water present in the sample, the pipes connecting the oven to the mass spectrometer are heated to a temperature of 50 °C. The oven is able to heat up to 1100 °C, but for this application, the temperature was limited to 200 °C to prevent the degradation of VOCs present in the sample.

#### 2.4.2. Sample Preparation

Two types of liquid samples were used in this study: (i) aqueous solutions of toluene with concentrations of a few mmol·L^−1^ for optimization and choice of the operating conditions, (ii) aqueous solution that containing a mixture of seven VOCs for testing system performances. The physicochemical properties of the seven VOCs are summarized in Table 1. The experiments with toluene solutions of 30 ppm up to 100 ppm toluene in water (*v*/*v*), i.e., 1.66 to 5.55 mmol·L^−1^ were prepared by dilution of pure toluene in 50 mL of milli-Q water. A new solution was prepared for each experiment. The mixtures of seven VOCs at different concentrations (300, 200, 150, 100, and 50 µg/mL for each COV) were prepared by mixing a few micro-liters of pure compounds in milli-Q water to obtain a final volume of 100 mL. Table 1 gives the properties of the compounds present in the mixture.

#### 2.4.3. Sample Vaporization Procedure

The non-woven gauze used was selected following preliminary studies showing that it emits no VOCs in the temperature range used, unlike other materials. The volumes of solution used vary from 0.1 to 1.0 mL according to the experiment. A 7.5 cm × 7.5 cm gauze is deposited on a ceramic combustion boat and soaked with the desired volume of VOC aqueous solution. The combustion boat is immediately placed at the center of the oven, the heat shields are put on, and the oven is closed. Dinitrogen circulation and mass spectra recording are immediately started. The analysis is continued until no VOC emission is detectable so that the entire deposited solution is expected to be vaporized. In this way, all VOCs present initially should be analyzed, with the exception of possible losses in surfaces.

#### 2.4.4. *Data Analysis and Analytes Quantification*

The data needed in order to quantify the VOCs present in the sample gas are the intensities of the different ions present on the mass spectra, the rate coefficients of the proton transfer reactions for the different VOCs, and the integrated value of the sample pressure over the reaction duration named (Pxt). It is measured by the mass spectrometer and expressed in 10^−4^ Torr·ms.

The mixing ratio X_Ai_ in ppm for each analyte A_i_ ionized by PTR is calculated using Equation (1):

(1) $$[\chi_{A_{i}}]=\frac{[\ln\left(\frac{Sum}{H_{H_{3}O^{+}}}\right)]\times [\sum_{j}F_{ij}^{+}]\times 10^{6}}{\left(Sum-H_{H_{3}O^{+}}\right)\times k_{A_{i}}\times \left(Pxt\right)\times 3.21}$$

where Sum is the sum of the absolute intensities of all ions, H_H3O_^+^ is the absolute intensity of hydronium ion, and Σ_j_F_ij_^+^ is the sum of the absolute intensities of all product ions resulting from the CI reaction of A_i_. k_Ai_ is the Proton Transfer Reaction (PTR) rate coefficient between the molecule A_i_ and the precursor H_3_O^+^. Pxt is the integrated value of the sample pressure in the ICR cell over reaction time t.

In most cases, the protonated ion A_i_H^+^ is the only product of the reaction of H_3_O^+^ with A_i_ and $\left[\sum_{j}F_{ij}^{+}]=[A_{i}H^{+}\right]$. Protonation of some compounds (such as alcohols) leads to fragmentation. In these cases, all fragments must be accounted for in order to give a correct quantification. In complex mixtures, where numerous fragmentations can be present, several compounds may have a common fragment, thus complicating the analysis. When this occurs, we choose one intense product ion generated exclusively from Ai and divide its intensity by its corresponding branching ratio noted α_i_. The branching ratios are known for the protonation reaction by H_3_O^+^ of many compounds and are reported in PTR-MS databases It is also possible to measure the branching ratios in our experimental conditions [38].

## 3. Results

### 3.1. Optimization and Choice of the Operating Conditions

Preliminary studies have been carried out to determine the best settings for the vaporization of VOCs in relation to our target application: sampling and analysis of sweat. Thus, the VOC samples chosen for this work are diluted in aqueous solutions deposited on a commonly used medical material: non-woven gauze. The influence of oven and sample parameters on the temporal signal are described in the following. The solution used for these initial tests is an aqueous solution of toluene. Toluene was chosen for these preliminary measurements because it is often used as a reference for the measurement of VOCs in the gas phase, and the calibration of the analyzer was previously verified using a calibration gas cylinder containing 20 ppm of toluene in N_2_.

#### 3.1.1. Temperature Ramp

**Figure 5:**
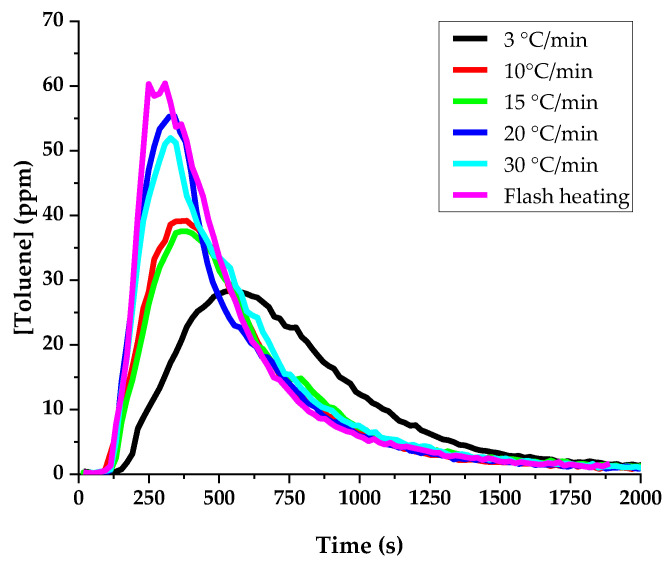
Toluene mixing ratio as a function of time in the gas flow when a gauze soaked with 0.5 mL of a 100 ppmv solution of toluene in water is heated using different temperature ramps at 20 mL/min N_2_ flow rate.

Different heating ramp rates were tested on a solution of 100 ppmv toluene in water. A gauze was soaked with 0.5 mL of the solution and immediately placed in the oven on a ceramic combustion boat. The sample is then heated from ambient temperature up to 200 °C. Six temperature ramp rates were tried: 3 °C/min, 10 °C/min, 15 °C/min, 20 °C/min, 30 °C/min, and flash heating, where the oven heats the sample from ambient temperature up to 200 °C at the fastest possible rate. The results are shown in Figure 5. The toluene mixing ratio in the gas at the exit of the oven is obtained from the measured ion intensities for protonated toluene and H_3_O^+^ using Equation (1) with a rate coefficient for the proton transfer reaction of 2.06 × 10^−9^ molecule^−1^·cm^3^·s^−1^ [39]. The observed time response for the mixing ratio of toluene is very dependent on the temperature ramp. The highest intensity is reached when using flash heating. With the slowest ramps, the response gets broader and extends toward longer times. As expected, the integrated mixing ratios, corresponding to the quantity of toluene present, are fairly constant (see Supplementary Materials Figure S1). After a few more experiments, we finally decided to use a ramp rate of 27 °C/min, which allowed us to have a good sensitivity with better control to reach the target temperature.

#### 3.1.2. Flow Rate of the Carrier Gas

**Figure 6:**
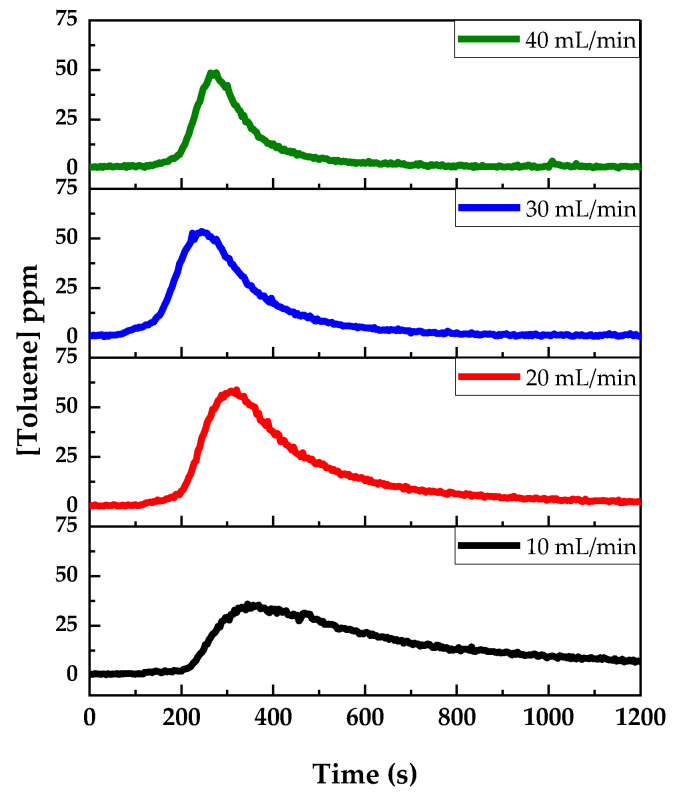
Influence of the carrier gas (N_2_) flow rate with a 0.5 mL of 100 ppmv toluene solution and a ramp of 27 °C/min.

The effect of the carrier gas flow rate on the response time was studied. As before, this study was performed using samples of gauze soaked with 0.5 mL of a 100 ppmv aqueous toluene solution. A temperature ramp of 27 °C/min was used. N_2_ and He were tried as non-reactive carrier gases with similar results, and we opted for N_2_, which is simpler to use and more available commercially. Figure 6 shows the results obtained with N_2_. The shape of the time dependence of the toluene mixing ratio varies strongly with the gas flow rate. With a low flow rate of 10 mL/min, the time response is larger: the toluene molecules diffuse in the buffer gas while they are carried slowly to the analyzer. At higher flow rates, the width of the time response peaks are shorter. The maximum in toluene intensity is obtained for a N_2_ flow rate of 20 mL/min, and for this reason, this was the flow rate chosen for the following experiments.

#### 3.1.3. Liquid Volume

**Figure 7:**
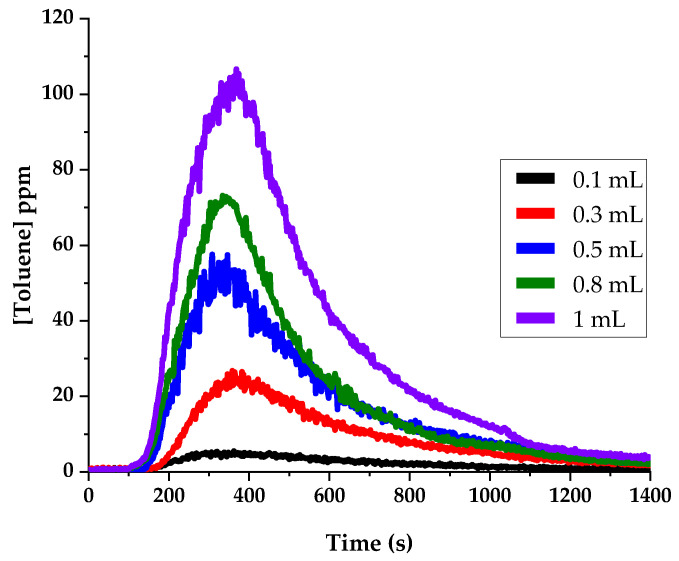
Influence of the liquid volume deposited on the non-woven gauze on the temporal profile observed for the mixing ratio of toluene in gas phase. Operating conditions: 0.5 mL of 100 ppmv toluene solution, 27 °C/min, 20 mL/min of N_2_ gas flow.

We carried out analyses with different volumes of the toluene 100 ppmv solution. The gauze was soaked with 0.1 mL, 0.3 mL, 0.5 mL, 0.8 mL, and 1 mL, and Figure 7 shows the toluene concentrations measured in the gas phase for the different volumes of liquid. The shapes of the time responses are similar, and the intensities increase proportionally with the liquid volume. Working with volumes of 0.5 mL appears as a good compromise, providing sufficient intensity with a reasonably small sample volume.

#### 3.1.4. Response at Different Toluene Concentrations: Linearity and Limit of Detection (LOD) for Toluene

**Figure 8:**
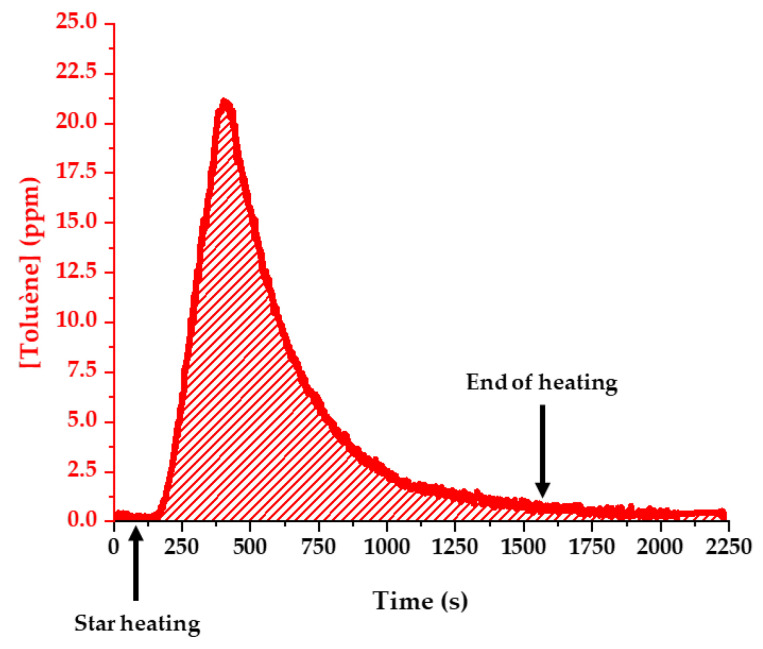
Evolution of toluene mixing ratios as a function of time. The area corresponding to the integration of the curve between 100 and 1600 ms is shown in hatching. Experimental conditions: 50 ppm toluene solution, 20 mL/min of N_2_, 27 °C/min.

The linearity of the response of the analytical instrument was checked for toluene using different concentrations (0, 30, 50, 80, and 100 ppmv in milli-Q water). The solution was prepared just before the experiment; a gauze was soaked with 0.5 mL and placed in the oven. Three different assays were performed on three different days for each toluene concentration. Thus, we have a set of three temporal records for the toluene mixing ratio, providing three calibration curves (details are given in Supplementary Materials). The ion intensities for protonated toluene are converted to a mixing ratio in the gas phase using Equation (1). Then, the temporal curves are integrated over the experiment duration time. Ion intensities are followed for 1600 s, and the heating ramp starts at t = 100 s. An example of the temporal evolution of the mixing ratio of a 50 ppm aqueous toluene solution is shown in Figure 8.

**Figure 9:**
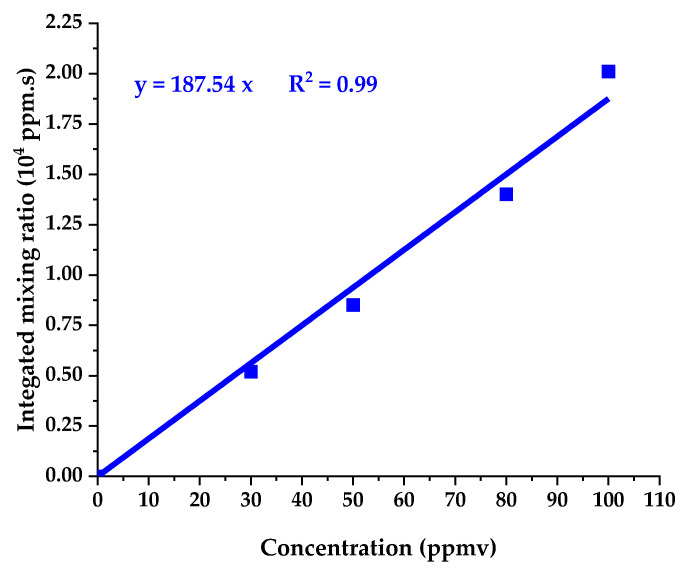
Mean integrated mixing ratio for toluene as a function of the toluene concentration in the solution.

The mean of integrated mixing ratios for the three assays is plotted in Figure 9 as a function of the toluene concentration in the initial aqueous solution. The response is expected to be proportional to the initial concentration for the four spiked samples. Figure 9 shows that this is indeed the case, despite the possible error causes, such as losses during the transfer on the tubing or heat shields.

The LOD is evaluated from the association of the measurement of the integrated toluene peak for several blank samples and the calibration curve. The blank sample measurements were performed on several days using gauze soaked with 0.5 mL of pure milli-Q water, which was introduced and heated the same way as for the samples containing toluene. The treatment was also performed similarly by measuring the peak intensity at *m*/*z* 93 as a function of time as well as the intensity of the precursor H_3_O^+^. Then, intensities were converted into mixing ratios using Equation (1), and these mixing ratios were integrated over time, starting at the beginning of the heat ramp and for a duration of 1500 s. Even if there is no toluene, the mixing ratios calculated are always non-zero because of the electrical noise that is always present in an FT-ICR measurement. A statistical analysis of the integrated mixing ratios obtained for the blanks gives their mean value M_093_ and their standard deviation S_093_.

A linear calibration curve such as

y = ax(2)

is fitted to the points shown in Figure 9 and gives a = 187.54.

The LOD is the initial concentration corresponding to an integrated mixing ratio equal to the mean value for the blanks added to three times the standard deviation:

LOD_093_ = (M_093_ + 3S_093_)/a (3)

In the case of toluene, we obtain M_093_ = 2053 ppm·s, S_093_ = 457 ppm·s, and LOD_093_ = 18 ppmv.

### 3.2. Analysis of a Mixture of VOCs

#### 3.2.1. Experimental Results and Comparison with Expected Mass Spectrum

The analytical performances of the instrument have been tested on an aqueous mixture of seven VOCs prepared in the laboratory, as explained in Section 2.4.2, containing methanol, ethanol, acetone, 2-butanone, 2-pentanone, 2-hexanone, and 1,4-dioxane diluted in water at different concentrations (300, 200, 150, 100, and 50 µg/mL) for each compound. These seven VOCs have been chosen so that none of them are isomers, and each of them reacts with H_3_O^+^ by simple proton transfer leading to AH^+^, either exclusively or predominantly. Thus, each of the product ions detected in the mixture can be clearly attributed to a single VOC.

We started our experiments by analyzing a solution containing 300 µg/mL of each VOC. A gauze soaked with 0.5 mL of this solution was placed in the oven and heated. A mass spectrum is recorded every three seconds. For these experiments, the heating ramp rate is 27 °C/min, and the N_2_ flow rate is 20 mL/min. The detected masses are shown in Table 2.

Table 2 gives the molecular formulas and *m*/*z* values of the detected product ions from the reaction of H_3_O^+^ with the mixture of seven VOCs, along with the expected *m/z* values and the corresponding exact mass differences. The reaction of H_3_O^+^ on methanol, ethanol, acetone, 2-butanone, 2-pentanone, and 2-hexanone gives only the protonated parent ions MH^+^ [21,37,40,41]. For 1,4-dioxane [21], some fragmentation is expected to occur and must be taken into account when performing the quantification.

The VOCs present in the solution are easily identified using the ion peaks present on the FT-ICR mass spectrum. The high precision allows the attribution of molecular formula to each peak.

In addition to measured ion intensities and Pxt, PTR rate coefficients are needed for quantification. With the H_3_O^+^ precursor, the rate coefficients are safely approximated by the capture rate coefficients. These can be evaluated from the molecular masses of ion and neutral reactants and from the neutral dipole moment and polarizability of the neutral [42,43]. Except for 2-hexanone, PTR rate coefficients for the six VOCs are already known. We evaluated the rate coefficient for 2-hexanone using its dipole moments and the polarizability of the neutral molecule. Its polarizability was evaluated using the Miller empirical method [44].

Table 3 shows the capture rate coefficients for the seven VOCs, the expected relative intensities derived from these rate coefficients, and the experimentally measured relative intensities for each VOC.

**Figure 10:**
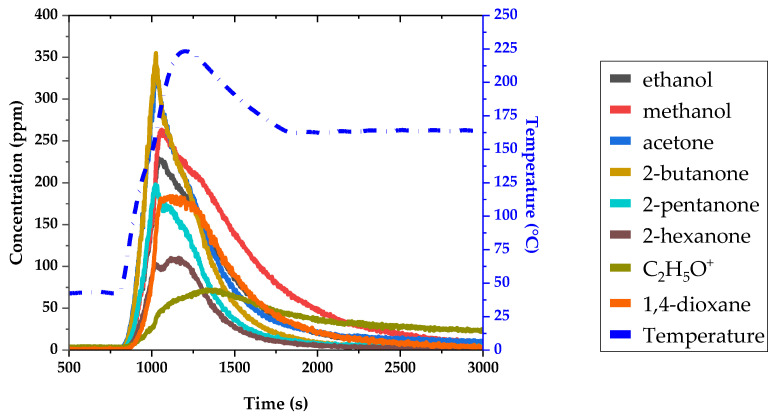
Intensity evolutions of a mixture of VOCs as a function of time.

The result of the real-time analysis showing the main ionic products observed as a function of time is given in Figure 10.

All seven VOCs are detected as their protonated ion. The corresponding temporal profiles are labeled in the figure. Another ionic product is detected: C_2_H_5_O^+^, at *m*/*z* 45.033, with a temporal profile very different from the seven other ones. This can be explained by the fact that this ion has two sources: (i) It is a fragment of protonated dioxane, and as its branching ratio is 20%, the corresponding intensity should be 20/80 of the protonated 1,4-dioxane intensity. (ii) This ion is also produced by the protonation of acetaldehyde, which is present as a permanent pollutant in different places of the experimental device. Therefore, the C_2_H_5_O^+^ ion was not considered for 1,4-dioxane quantification, which was based only on C_4_H_9_O_2_^+^ using the corresponding branching ratio.

The different VOCs present are expected to have different vaporization behaviors depending on their pressure vapor, solubility, and partition coefficient between the liquid and gas phases. Therefore, the temporal profiles for each compound are somewhat different. Figure 10 shows the temporal profiles of the mixing ratio recorded for each VOC. The time responses reflect the speed at which each VOC is vaporized.

The initial steep rise in concentration and the shape of the maximum zone show the successive appearance of groups of VOCs: acetone and 2-butanone are emitted first; then 2-pentanone, methanol, and ethanol; and finally, 1,4-dioxane and 2-hexanone. For all these compounds, the shapes of the concentration decrease profiles are similar, except for methanol and, to a lesser extent, acetone, which last longer over time. Even after one hour of heating, a small amount remains in the gas phase for these compounds. This could be due to adsorption on different surfaces of the instrument and tubings followed by slow release.

**Figure 11:**
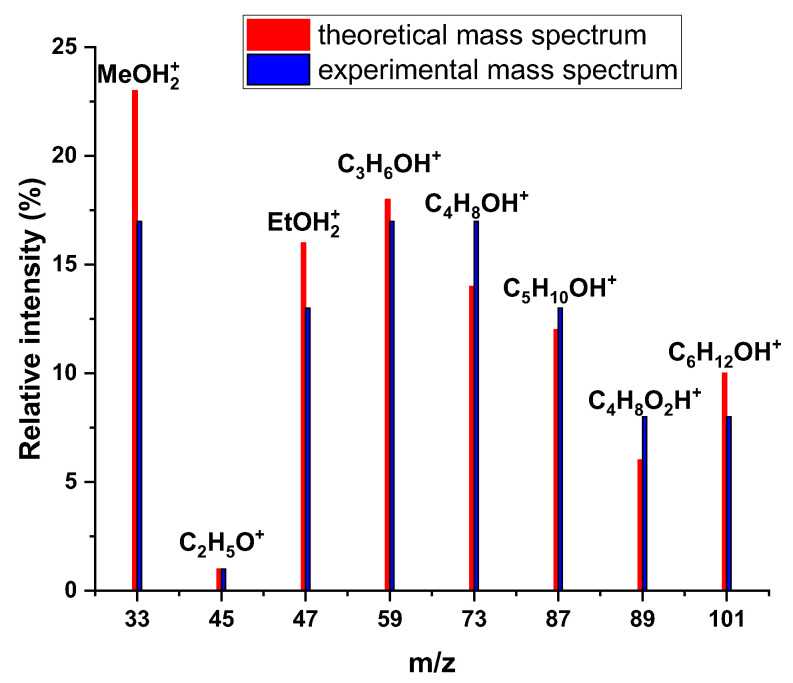
Mass spectrum obtained after the analysis of the standard mixture of VOCs. The concentrations of compounds were 300 µg/mL.

The experimental mass spectrum averaged over the duration of the analysis for 0.5 mL of aqueous solution containing 300 µg/mL of each of the seven VOCs is given in Figure 11. It is compared with a calculated mass spectrum, derived using the initial quantity present initially for each of the VOCs and the parameters characterizing their chemical ionization (rate coefficient of the reaction with H_3_O^+^ and, when necessary, the branching ratio for the different ionic products). The resulting ion relative intensities for both the experimental and calculated mass spectra are shown in Figure 11.

All the expected ions are present on the mass spectrum. As expected from the literature [21], only 1,4-dioxane gives a fragment ion. Figure 11 shows a good agreement between the theoretical and experimental mass spectra: the measured and the calculated relative intensities are very similar for all compounds, with slight overestimation of methanol, ethanol, and hexan-2-one and underestimation of butanone and 1,4-dioxane.

#### 3.2.2. Evaluation of Instrument Response and LOD Determination for the Seven VOCs

**Figure 12:**
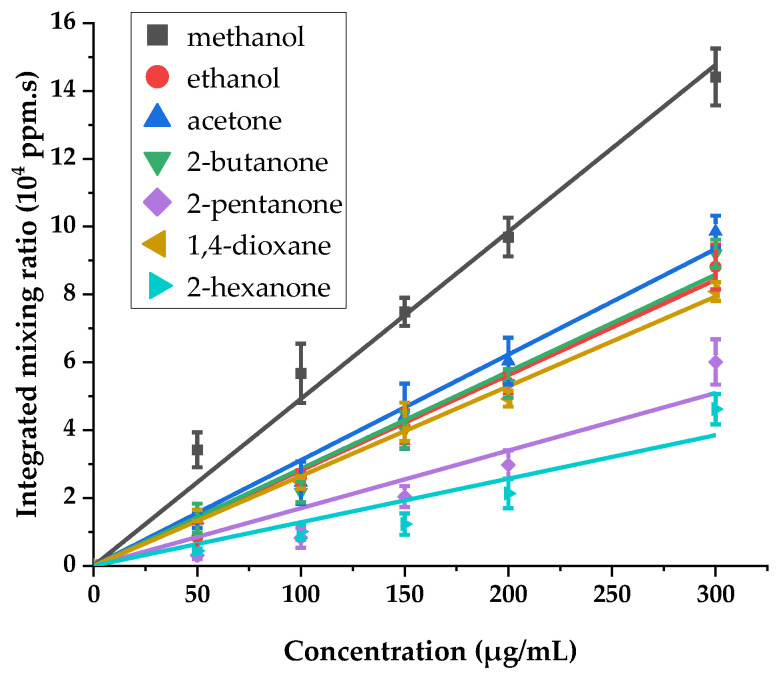
Response of MS/tubular oven to increasing concentrations of VOCs.

The mixture of seven VOCs was prepared at different concentrations (50, 100, 150, 200, and 300 µg/mL in milli-Q water for each compound) to test the linearity of the instrument’s response. All solutions were prepared just before the experiment. For each concentration, three assays were performed. As with the method used to determine the LOD for toluene, the ion intensities for each VOC of the mixture were converted to the mixing ratio in the gas phase using the quantification equation (Equation (1)). Then, the mixing ratio curves were integrated over the experiment duration time to obtain areas under curves. We report in Figure 12 the integrated mixing ratio for each compound as a function of its initial concentration in aqueous solutions.

As shown in Figure 12, the response of the instrument with increasing concentrations is reasonably linear for all compounds present in the mixture. More details on the linear fit of the experimental result are given in the Supplementary Materials. The correlation coefficients R^2^ of the linear regressions are higher than 0.98 for methanol, ethanol, acetone, 2-butanone, 1,4-dioxane, and 2-hexanone. For 2-pentanone, the obtained integrated mixing ratios are somewhat more spread away from the linear fit, leading to a lower correlation coefficient R^2^ of 0.95. This dispersion may be explained by the low volatility of this molecule, which can cause its adsorption on the internal surface of the quartz tube and on the transfer line between the oven and the mass spectrometer.

In order to determine the LOD, we analyzed several blanks using a sample of pure milli-Q water. Just as described before with toluene, the data treatment was carried out to obtain the intensities at the *m*/*z* value of each VOC together with the H_3_O^+^ intensities. Then, mixing ratios were calculated using Formula (1) and integrated over time.

A statistical analysis gives the mean value of the blanks and the standard deviation for each VOC. The LODs are then calculated using the formula:

(4) $${LOD}_{VOC}=(M_{VOC}+3S_{VOC})/a_{VOC}$$

The LODs obtained are 26 ppm for methanol, 32 ppm for ethanol, 42 ppm for acetone, 15 ppm for 2-butanone, 24 ppm for 2-pentanone, 5 ppm for 1,4-dioxane, and 8 ppm for 2-hexanone.

## 4. Conclusions

In this paper, we present a new instrumental coupling in which a tubular oven is associated with a CI-FT-ICR mass spectrometer with the objective of characterizing VOCs present in small samples of liquid. The device provides quantitative information concerning VOCs present in aqueous samples. The tubular oven is used to heat the aqueous sample and transfer both water and VOCs of interest in the gas phase. The vaporized species are transported by an optimized stream of N_2_ carrier gas to the mass spectrometer, where they are analyzed. Thanks to the selectivity of CI, only VOCs are ionized. The permanent magnet FT-ICR mass spectrometer monitors the VOCs emitted in the gas phase when the sample is heated. Its good mass resolution allows the identification of compounds present in the aqueous solution. Because the chemical ionization is performed in well-defined pressure conditions, the VOCs are quantified at each acquisition point and give a dynamic view of the VOCs’ evolution in the gas phase. With our method, the observed concentrations of the VOCs in the gas phase are integrated over time, and the resulting measurements show a linear response with respect to the initial concentration of the VOCs in the liquid samples. Finally, the method described allows the analysis of a complex mixture of VOCs and features good repeatability and reproducibility. For the different VOCs studied, the LOD is in the 5–50 ppm range. LODs can be improved by averaging the signals. Reducing the volume of the oven chamber could also improve LODs and reduce the duration of the analysis.

## Figures and Tables

**Table 1:** Physico-chemical properties of the VOCs present in the standard mixture [21,36,37].

Compound	Molecular Formula	Molecular Weight (g·mol^−1^)	Vapor Pressure (Pa) at 20 °C	Dipole Moment (D)	Proton Affinity (kJ·mol^−1^)
Methanol	CH_4_O	32.04	13,023	1.70	754.3
Ethanol	C_2_H_6_O	46.08	5786	1.69	776.4
Acetone	C_3_H_6_O	58.08	24,598	2.88	812.0
2-butanone	C_4_H_8_O	72.11	9466	2.78	827.3
2-pentanone	C_5_H_10_O	86.13	1600	2.70	832.7
1,4-dioxane	C_4_H_8_O_2_	88.11	3861	0	797.4
2-hexanone	C_6_H_12_O	100.16	516	2.66	843.2

**Table 2:** Detected ion products and comparison of observed and expected *m*/*z* values.

Compound	Expected/Detected Product Ions(s)	Detected *m*/*z*	Expected *m*/*z*	Δm × 10^−3^ (u) **
Methanol	CH_5_O^+^	33.0350	33.0335	+1.5
Ethanol	C_2_H_7_O^+^	47.0508	47.0491	+1.7
Acetone	C_3_H_7_O^+^	59.0505	59.0491	+1.4
2-butanone	C_4_H_9_O^+^	73.0650	73.0648	+0.2
2-pentanone	C_5_H_11_O^+^	87.0798	87.0804	-0.6
1,4-dioxane *	C_4_H_9_O_2_^+^ C_2_H_5_O^+^	89.0577 (0.80); 45.0347 (0.20)	89.0597 45.0335	−2.0 +1.2
2-hexanone	C_6_H_13_O^+^	101.0922	101.0961	−3.9

* In parentheses: branching ratio of each channel. ** Δm is defined as Detected *m*/*z* − Expected *m*/*z*.

**Table 3:** Comparison of the experimental and expected intensities for the peaks present on the mass spectrum.

VOC	Capture Rate Coefficients (10^−9^ cm^3^·s^−1^)	Experimental Relative Intensities of Ions for 300 µg·mL^−1^ Solution (%)	Expected Relative Intensities of Ions for 300 µg·mL^−1^ Solution (%)
Methanol	2.30	17	23
Ethanol	2.31	13	16
Acetone	3.23	17	18
2-butanone	3.21	17	14
2-pentanone	3.19	13	12
1,4-dioxane	1.80	8	6
2-hexanone	3.19 *	8	10

* The capture rate coefficients were determined from ion and neutral masses, dipole moments, and polarizabilities of the neutrals. Polarizabilities were calculated using the Miller method.

## Data Availability

Data are contained within the article and Supplementary Materials.

## Supplementary Materials

The following supporting information can be downloaded at: https://www.mdpi.com/article/10.3390/s24041048/s1, Table S1: Detailed timing of the FT-ICR analytical sequence; Figure S1: Integrated mixing ratio of toluene as a function of temperature ramp rate; Figure S2: The three calibration curves for toluene; Table S2: Integrated mixing ratio values of toluene in the gas phase for each concentration of toluene in the solution for three different assays. Table S3: Mean integrated mixing ratio values and standard deviations S for the 7 VOCs; Table S4: Linear regression for the 7 VOCs: equations and correlation coefficients.

## References

[1] Kuráň P., Soják L. (1996). Environmental Analysis of Volatile Organic Compounds in Water and Sediment by Gas Chromatography. J. Chromatogr. A.

[2] Huybrechts T., Dewulf J., Moerman O., Van Langenhove H. (2000). Evaluation of Purge-and-Trap-High-Resolution Gas Chromatography-Mass Spectrometry for the Determination of 27 Volatile Organic Compounds in Marine Water at the Ng l^−1^ Concentration Level. J. Chromatogr. A.

[3] Dewulf J., Van Langenhove H., Wittmann G. (2002). Analysis of Volatile Organic Compounds Using Gas Chromatography. TrAC Trends Anal. Chem..

[4] Yang X., Wang C., Shao H., Zheng Q. (2019). Non-Targeted Screening and Analysis of Volatile Organic Compounds in Drinking Water by DLLME with GC–MS. Sci. Total Environ..

[5] Cavalcante R.M., de Andrade M.V.F., Marins R.V., Oliveira L.D.M. (2010). Development of a Headspace-Gas Chromatography (HS-GC-PID-FID) Method for the Determination of VOCs in Environmental Aqueous Matrices: Optimization, Verification and Elimination of Matrix Effect and VOC Distribution on the Fortaleza Coast, Brazil. Microchem. J..

[6] Ikem A. (2010). Measurement of Volatile Organic Compounds in Bottled and Tap Waters by Purge and Trap GC–MS: Are Drinking Water Types Different?. J. Food Compos. Anal..

[7] Niri V.H., Bragg L., Pawliszyn J. (2008). Fast Analysis of Volatile Organic Compounds and Disinfection By-Products in Drinking Water Using Solid-Phase Microextraction–Gas Chromatography/Time-of-Flight Mass Spectrometry. J. Chromatogr. A.

[8] Teske J., Efer J., Engewald W. (1998). Large-Volume PTV Injection: Comparison of Direct Water Injection and in-Vial Extraction for GC Analysis of Triazines. Chromatographia.

[9] Teske J., Efer J., Engewald W. (1997). Large-Volume PTV Injection: New Results on Direct Injection of Water Samples in GC Analysis. Chromatographia.

[10] Demeestere K., Dewulf J., De Witte B., Van Langenhove H. (2007). Sample Preparation for the Analysis of Volatile Organic Compounds in Air and Water Matrices. J. Chromatogr. A.

[11] Allers M., Langejuergen J., Gaida A., Holz O., Schuchardt S., Hohlfeld J.M., Zimmermann S. (2016). Measurement of Exhaled Volatile Organic Compounds from Patients with Chronic Obstructive Pulmonary Disease (COPD) Using Closed Gas Loop GC-IMS and GC-APCI-MS. J. Breath Res..

[12] Bedner M., Saito K. (2020). Development of a Liquid Chromatography Atmospheric Pressure Chemical Ionization Mass Spectrometry Method for Determining Off-Flavor Compounds and Its Application toward Marine Recirculating Aquaculture System Monitoring and Evaluation of Aeration as a Depuration Approach. J. Chromatogr. A.

[13] Du B., Shen M., Pan Z., Zhu C., Luo D., Zeng L. (2022). Trace Analysis of Multiple Synthetic Phenolic Antioxidants in Foods by Liquid Chromatography–Tandem Mass Spectrometry with Complementary Use of Electrospray Ionization and Atmospheric Pressure Chemical Ionization. Food Chem..

[14] Calderón-Santiago M., Priego-Capote F., Jurado-Gámez B., Luque De Castro M.D. (2014). Optimization Study for Metabolomics Analysis of Human Sweat by Liquid Chromatography–Tandem Mass Spectrometry in High Resolution Mode. J. Chromatogr. A.

[15] Delgado-Povedano M.M., Castillo-Peinado L.S., Calderón-Santiago M., Luque De Castro M.D., Priego-Capote F. (2020). Dry Sweat as Sample for Metabolomics Analysis. Talanta.

[16] Delgado-Povedano M.M., Calderón-Santiago M., Priego-Capote F., Luque De Castro M.D. (2016). Development of a Method for Enhancing Metabolomics Coverage of Human Sweat by Gas Chromatography–Mass Spectrometry in High Resolution Mode. Anal. Chim. Acta.

[17] Delgado-Povedano M.M., Calderón-Santiago M., Luque De Castro M.D., Priego-Capote F. (2018). Metabolomics Analysis of Human Sweat Collected after Moderate Exercise. Talanta.

[18] Sun L., Qi Y., Meng M., Cui K. (2023). Comparative Study on the Volatile Organic Compounds and Characteristic Flavor Fingerprints of Five Varieties of Walnut Oil in Northwest China Using Using Headspace Gas Chromatography-Ion Mobility Spectrometry. Molecules.

[19] Lindinger W., Jordan A. (1998). Proton-Transfer-Reaction Mass Spectrometry (PTR–MS): On-Line Monitoring of Volatile Organic Compounds at Pptv Levels. Chem. Soc. Rev..

[20] Ellis A.M., Mayhew C.A. (2013). Proton Transfer Reaction Mass Spectrometry: Principles and Applications.

[21] Lindinger W., Hansel A., Jordan A. (1998). On-Line Monitoring of Volatile Organic Compounds at Pptv Levels by Means of Proton-Transfer-Reaction Mass Spectrometry (PTR-MS) Medical Applications, Food Control and Environmental Research. Int. J. Mass Spectrom. Ion Process..

[22] Tanimoto H., Aoki N., Inomata S., Hirokawa J., Sadanaga Y. (2007). Development of a PTR-TOFMS Instrument for Real-Time Measurements of Volatile Organic Compounds in Air. Int. J. Mass Spectrom..

[23] Cappellin L., Biasioli F., Fabris A., Schuhfried E., Soukoulis C., Märk T.D., Gasperi F. (2010). Improved Mass Accuracy in PTR-TOF-MS: Another Step towards Better Compound Identification in PTR-MS. Int. J. Mass Spectrom..

[24] Mauclaire G., Lemaire J., Boissel P., Bellec G., Heninger M. (2004). MICRA: A Compact Permanent Magnet Fourier Transform Ion Cyclotron Resonance Mass Spectrometer. Eur. J. Mass Spectrom..

[25] Heninger M., Mestdagh H., Louarn E., Mauclaire G., Boissel P., Leprovost J., Bauchard E., Thomas S., Lemaire J. (2018). Gas Analysis by Electron Ionization Combined with Chemical Ionization in a Compact FTICR Mass Spectrometer. Anal. Chem..

[26] Lemaire J., Thomas S., Lopes A., Louarn E., Mestdagh H., Latappy H., Leprovost J., Heninger M. (2018). Compact FTICR Mass Spectrometry for Real Time Monitoring of Volatile Organic Compounds. Sensors.

[27] Louarn E., Asri-Idlibi A.M., Leprovost J., Héninger M., Mestdagh H. (2018). Evidence of Reactivity in the Membrane for the Unstable Monochloramine during MIMS Analysis. Sensors.

[28] Louarn E., Hamrouni A., Colbeau-Justin C., Bruschi L., Lemaire J., Heninger M., Mestdagh H. (2013). Characterization of a Membrane Inlet Interfaced with a Compact Chemical Ionization FT-ICR for Real-Time and Quantitative VOC Analysis in Water. Int. J. Mass Spectrom..

[29] Roumiguières A., Kinani S., Bouchonnet S. (2020). Tracking Monochloramine Decomposition in MIMS Analysis. Sensors.

[30] Malfondet N., Brunerie P., Le Quéré J.-L. (2021). Discrimination of French Wine Brandy Origin by PTR-MS Headspace Analysis Using Ethanol Ionization and Sensory Assessment. Anal. Bioanal. Chem..

[31] Romano A., Capozzi V., Khomenko I., Biasioli F. (2023). Advances in the Application of Direct Injection Mass Spectrometry Techniques to the Analysis of Grape, Wine and Other Alcoholic Beverages. Molecules.

[32] Fiches G., Déléris I., Saint-Eve A., Brunerie P., Souchon I. (2014). Modifying PTR-MS Operating Conditions for Quantitative Headspace Analysis of Hydro-Alcoholic Beverages. 2. Brandy Characterization and Discrimination by PTR-MS. Int. J. Mass Spectrom..

[33] Aprea E., Biasioli F., Märk T.D., Gasperi F. (2007). PTR-MS Study of Esters in Water and Water/Ethanol Solutions. Int. J. Mass Spectrom..

[34] Karl T., Yeretzian C., Jordan A., Lindinger W. (2003). Dynamic Measurements of Partition Coefficients Using Proton-Transfer-Reaction Mass Spectrometry (PTR–MS). Int. J. Mass Spectrom..

[35] Liu D., Feilberg A., Nielsen A.M., Adamsen A.P.S. (2013). PTR-MS Measurement of Partition Coefficients of Reduced Volatile Sulfur Compounds in Liquids from Biotrickling Filters. Chemosphere.

[36] Buhr K., van Ruth S., Delahunty C. (2002). Analysis of Volatile Flavour Compounds by Proton Transfer Reaction-Mass Spectrometry: Fragmentation Patterns and Discrimination between Isobaric and Isomeric Compounds. Int. J. Mass Spectrom..

[37] Kajos M.K., Rantala P., Hill M., Hellén H., Aalto J., Patokoski J., Taipale R., Hoerger C.C., Reimann S., Ruuskanen T.M. (2015). Ambient Measurements of Aromatic and Oxidized VOCs by PTR-MS and GC-MS: Intercomparison between Four Instruments in a Boreal Forest in Finland. Atmos. Meas. Tech..

[38] Latappy H., Lemaire J., Heninger M., Louarn E., Bauchard E., Mestdagh H. (2016). Protonated 1,4-Difluorobenzene C6H5F2+: A Promising Precursor for Proton-Transfer Chemical Ionization. Int. J. Mass Spectrom..

[39] Gueneron M., Erickson M.H., VanderSchelden G.S., Jobson B.T. (2015). PTR-MS Fragmentation Patterns of Gasoline Hydrocarbons. Int. J. Mass Spectrom..

[40] Karl T.G., Christian T.J., Yokelson R.J., Artaxo P., Hao W.M., Guenther A. (2007). The Tropical Forest and Fire Emissions Experiment: Method Evaluation of Volatile Organic Compound Emissions Measured by PTR-MS, FTIR, and GC from Tropical Biomass Burning. Atmos. Chem. Phys..

[41] Španěl P., Pavlik M., Smith D. (1995). Reactions of H3O+ and OH− Ions with Some Organic Molecules; Applications to Trace Gas Analysis in Air. Int. J. Mass Spectrom. Ion Process..

[42] Su T., Chesnavich W.J. (1982). Parametrization of the Ion–Polar Molecule Collision Rate Constant by Trajectory Calculations. J. Chem. Phys..

[43] Pagonis D., Sekimoto K., de Gouw J. (2019). A Library of Proton-Transfer Reactions of H3O+ Ions Used for Trace Gas Detection. J. Am. Soc. Mass Spectrom..

[44] Miller K.J. (1990). Additivity Methods in Molecular Polarizability. J. Am. Chem. Soc..

