# Supplementary material for: Analysis of VOCs in Liquids through Vaporization in a Tubular Oven Monitored by Chemical Ionization Mass Spectrometry
Source: Sensors (Basel). 2024 Feb 6;24(4):1048. doi: 10.3390/s24041048 (PMC10891908; doi:10.3390/s24041048)
Supplement: Supplementary file 1 [file sensors-24-01048-s001.zip › sensors-2813699-supplementary.pdf]

Supplementary material

Analysis of VOCs in liquids through vaporization in a tubular oven monitored by Chemical Ionization Mass Spectrometry

Taous Abar [1], Hélène Mestdagh [1], Michel Heninger [1,*] and Joel Lemaire [1]

[1] Institut de Chimie Physique, CNRS, Université Paris-Saclay
* Correspondence: michel.heninger@universite-paris-saclay.fr; Tel.: +33 1 69 15 34 58 (M.H.);

Table S1: Detailed timing of the FT-ICR analytical sequence

	Action	Action explanation	Beginning time (ms)	Action duration (ms)
**1**	quench	Ejection of all ions from the cell	10.0	50.0
**2**	H_2O	Water vapor introduction	100.0	10.0
**3**	electrons	Introduction of electrons via intermediate electrode	140	10.0
**4**	excit ejectall	Ejection of all ions except H_3O^+	450.0	1.0
**5**	piezo3	Piezoelectric valve opening: sample gas introduction	600.0	150
**6**	detect full	Ion excitation and detection of all ions	1600.0	1.1
**7**	loop	Repetition of the sequence according to data acquisition program	2500.0	

Table S1 gives the timing sequence used with the FT-ICR mass spectrometer. The different actions are done sequentially and are repeated when the last line (loop) is reached. Water vapor gas is introduced on line two and is followed by the electron beam to produce the H_3O^+ ions. Then, after the ejection of unwanted ions, the sample gas is introduced on line 5, and some time is left before the excitation/detection event so that the pressure in the ICR is low enough for FT-ICR detection.

Figure S1: Integrated mixing ratio of toluene as a function of temperature ramp rate

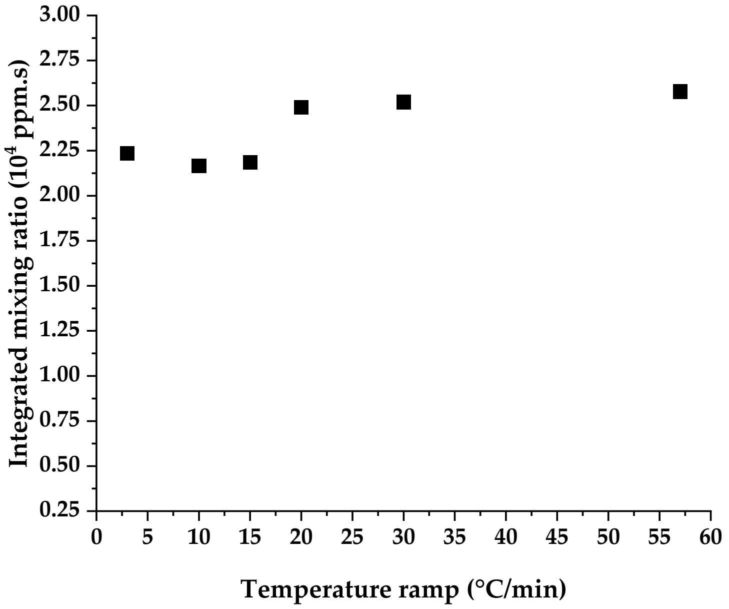

The effect of different temperature ramps was tested using gauze soaked with 0.5 ml of a 100 ppmv solution of toluene in water. For each temperature ramp of the oven, the mixing ratio of toluene in the gas phase was recorded as a function of time and the resulting curve was integrated, starting at the beginning of the ramp and for a duration of 1500 s.

Figure S1 shows the different integrated values obtained as a function of the different temperature ramps tested. The last one corresponds to the flash heating mode, for which the slope corresponds to a 57°/min heating (however, in this mode, we observe a temperature overshoot before reaching the final temperature).

We can see that the different integrated values are quite close: the total quantity of toluene observed in the gas phase is almost the same for the different temperature ramps used.

Figure S2: The three calibration curves for toluene

Figure S2 shows the results obtained when measuring the integrated mixing ratio of toluene in the gas phase at different concentrations. The three assays have been done on three different days. Here, a calibration curve is obtained for each assay by fitting a linear curve y=ax to the point obtained at different toluene concentrations in the solution.

The equation of the fitting curves is given in the figure. The values of the integrated mixing ratio for the different toluene concentrations in the solution are given in Table S2

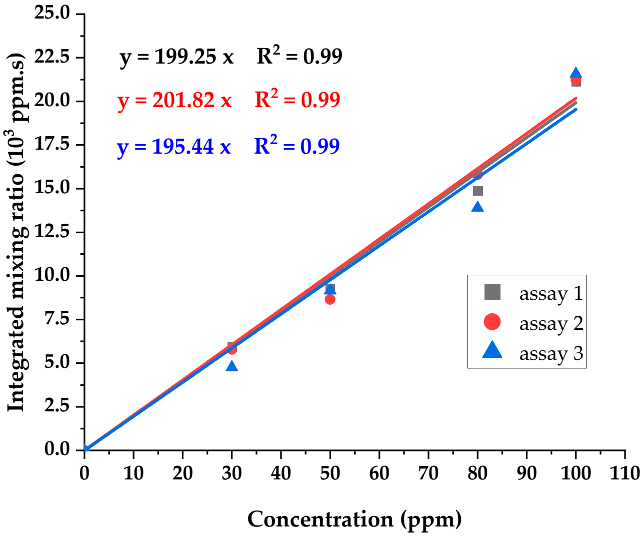

Table S2: Integrated mixing ratio values of toluene in the gas phase for each concentration of toluene in the solution for three different assays

[C]	Assay 1	Assay 2	Assay 3
ppm	ppm*s		
100	21126	21246	21574
80	14885	15811	13898
50	9282	8654	9155
30	5920	5792	4761

Table S3: Mean integrated mixing ratio values and standard deviations S for the 7 VOCs

The following table gives the integrated mixing ratio for the 7 VOCs as observed in the gas phase when these VOCs are present in the water solution at concentrations between 50 and 300 µg/ml.

The mixing ratios are obtained using the ion intensities on the mass spectra, the value of Pxt (pressure integrated, ensuring the introduction of the sample gas), and the rate coefficient for the protonation reaction by H_3O^+. They are integrated over time, starting at the beginning of the temperature ramp.

[C]	MeOH	S	EtOH	S	acetone	S	2-butanone	S	2-pentanone	S	1,4-dioaxane	S	2-hexanone	S
µg/mL	ppm.s													
300	144137	8409	88123	6632	98552	4669	92006	4157	60114	6701	80858	2805	63366	6727
200	96935	5706	53922	2933	60360	6877	53746	4165	29746	4258	49248	2351	32484	5560
150	74882	4153	40960	4860	44095	9609	39953	5324	20401	3092	42395	5684	21124	8217
100	56758	8738	25969	2156	24503	6235	21680	2985	8249	2949	24591	1841	18288	3610
50	34190	5158	8348	4358	13460	2277	14043	4261	3087	1151	14856	1641	11190	870

Table S4: Linear regression for the 7 VOCs: equations and correlation coefficients

For each VOC the points obtained (integrated mixing ratio as a function of the initial concentration in the liquid) were fitted by a linear curve Y=ax. The equation of these curves and the corresponding correlation coefficients are given here:

MeOH 492.40x $R^2 = 0.99$

EtOH 281.09x $R^2 = 0.99$

Acetone 311.37x $R^2 = 0.99$

2-butanone 286.15x $R^2 = 0.99$

2-pentanone 169.84x $R^2 = 0.95$

1,4-dioxane 264.66x $R^2 = 0.99$

2-hexanone 188.26x $R^2 = 0.98$

Theoretical mass spectrum estimation for Figure 11

To calculate the theoretical mass spectrum for the mixture of the 7 VOCs, first, we calculated the mole number of each VOC present in the 0.5 mL of the 300 μg/mL mixture solution. Considering the nitrogen flow rate (20 mL/min) and the duration time of the analysis, we converted these mole numbers to ppm. By multiplying the obtained value by the PTR capture rate coefficient rate and the branching ratio of each VOC, we estimate the intensity of each VOC of the mixture.
